# Functional and perfusion changes associated with silicone oil tamponade after macula-off rhegmatogenous retinal detachment surgery: an optical coherence tomography angiography/microperimetry study

**DOI:** 10.1007/s10792-024-03037-5

**Published:** 2024-02-22

**Authors:** Ghada A. Nassar, Hebatalla Samir Makled, Maha Mohamed Youssef, Lameece Moustafa Hassan

**Affiliations:** Ophthalmology Department, Kasr Al Ainy Hospital, Faculty of Medicine, Cairo University, Cairo, 11562 Egypt

**Keywords:** Optical coherence tomography angiography, Retinal microvasculature, Retinal sensitivity, Silicone oil removal, Optic nerve head vasculature, Microperimetry

## Abstract

**Purpose:**

The current study utilizes microperimetry and optical coherence tomography angiography (OCTA) to assess the optic nerve head vasculature, retinal microvasculature, and retinal sensitivity before and after silicone oil (SO) removal.

**Methods:**

This prospective observational case series study involved 30 eyes subjected to silicone oil endotamponade. Microperimetry and OCTA were utilized to assess the vascular density (VD) of the macula and optic nerve head, as well as the retinal sensitivity (RS), of the participants preoperatively and 1 month following SO removal. The correlation between the various parameters of OCTA and microperimetry was evaluated.

**Results:**

There was a significant improvement in the postoperative best-corrected visual acuity (BCVA) (*p-*value < 0.001) and the postoperative total RS, which was 6.38 ± 2.34 dB as compared to a mean preoperative total RS of 5.04 ± 2.06 dB (*p*-value < 0.001) and showing a significant increase in all rings. However, there was no significant difference in the pre and postoperative macular VD. On the other hand, there was a significant increase in the postoperative VD of the whole disk and the peripapillary capillary plexus, *p*-values < 0.001 and 0.002*,* respectively.

**Conclusion:**

The removal of SO resulted in significant improvements in retinal sensitivity, vision, and optic nerve perfusion. However, no significant change was observed in macular VD.

Clinical Trials.gov Identifier: NCT04928196.

## Introduction

While the surgical success rate of pars plana vitrectomy (PPV) with SO tamponade for rhegmatogenous retinal detachment (RRD) is approximately 85%, the postoperative visual prognosis remains unpredictable, and anatomical success does not always guarantee favorable functional outcomes [1–4].

Several studies investigated the underlying cause of visual loss even after the foveal structure has been restored following successful vitrectomy, particularly after the utilization of SO. The proposed mechanisms suggest that the optic nerve damage and thinning of retinal layers occur due to the direct infiltration of SO into the tissue, as well as its potential toxicity on the horizontal and bipolar cells [5, 6].

Similarly, it has been demonstrated that SO tamponade can impact and potentially alter retinal and choroidal microstructures [7]. The process can be assessed using OCTA, a non-invasive vascular imaging approach for visualization of the retinal microvasculature without dye leakage. OCTA allows for layer-by-layer analysis and provides imaging of the deeper vascular zones. Additionally, it enables evaluation of the foveal avascular zone (FAZ) and vessel density (VD) areas, retinal nerve fiber layer thickness (RNFLT), and peripapillary VD [8–11].

Microperimetry of the macula is a fundus-tracking controlled visual field examination that precisely determines the relationship between morphological alterations and functional faults. It has been utilized as a valuable diagnostic tool for the evaluation of the macular function in SO-filled eyes [12–15].

In our study, we evaluated the optic nerve head and macular vasculature by OCTA and retinal sensitivity using microperimetry, both before and after the removal of SO, allowing us to correlate the structural and functional outcomes of these patients.

## Materials and methods

In this prospective observational case series study, 30 individuals were examined before and after SO removal.

It was conducted in the Cairo University Hospital’s Ophthalmology Department from September 2021 to 2022 in accordance with the Helsinki Declaration’s tenets. Prior to enrollment in the trial, each patient provided a written consent form approving their participation and data release.

For rhegmatogenous macula-off retinal detachment, all cases had successful 23-gauge pars plana vitrectomy with SO injection (BIOSIL silicone oil 5000 centistokes by OMNIA Fluid, Italy). Cases of both genders aged from 18–60 years were included. We excluded patients with any form of maculopathy, such as diabetic maculopathy or proliferative diabetic retinopathy, choroidal neovascularization, macular scar, or chorioretinal degeneration involving the macula. Furthermore, cases of optic neuropathy, such as glaucomatous optic nerve damage, were also excluded. All cases of recurrent detachment or macular hole were excluded. In the presence of secondary complications, such as cataracts, SO emulsification, or secondary glaucoma/hypotony, the patient was excluded and replaced. Likewise, we excluded cases in which media opacities impeded the acquisition of high-quality imaging.

The patients underwent complete ophthalmological assessment preoperatively (2 days prior to silicone removal) and postoperatively (1-month postoperative). The pre- and post-silicone removal imaging included:

- *OCTA images* were obtained utilizing the Avanti RTVue system (Optovue Inc, Fremont, CA, USA). These images included an optic disk angiogram measuring 4.5 × 4.5 mm aligned into the disk and macular 6 × 6 mm scans with AngioVUe 3D projection artifact removal. The OCTA machine software was used to calculate the vascular density in both DCP and SCP and choriocapillaris.
- *OCT scans* of the retinal nerve fiber layer (RNFL) and macula were obtained with the same Optovue machine using radial lines and a high-definition scan.
- *Microperimetry* was used to assess retinal sensitivity utilizing the OPTOS Spectral OCT/SLO (scanning laser ophthalmoscope) (OPTOS, Inc., FL, USA). Patients maintained central fixation on a red target with mildly dilated pupils following a 20-min period of dark adaptation. Any refractive error was automatically corrected by the machine. A customized pattern centered on 11° was used, featuring the size of Goldmann III, 200-ms duration, and 1500-ms interval between stimuli. The 28 points of retinal sensitivity were measured with the inner ring at 2.3°, the middle ring at 6.6°, and the outer ring at 11° with levels ranging from 0 to 20 dB. The total retinal sensitivity and sensitivity of each layer were assessed.

### Surgical steps of silicone oil removal

One surgeon, for all cases, used a two-port (infusion-extraction) method, where two conventional sclerotomies were made across the temporal pars plana using a 23-gauge silicone injection/aspiration cannula.

### Statistical analysis

Data were analyzed and described using frequency and proportion for qualitative variables, mean values, standard deviations or medians, and variations for continuous variables based on normality. Changes before and after treatment were evaluated using the Wilcoxon signed-rank test or paired t-tests, depending on normality. Correlations were assessed using the Spearman rank correlation equation.

## Results

### Epidemiology and clinical data

The study involved 30 eyes from 30 individuals, with an average age of 41.87 ± 11.54 years (ranging from 22 to 60). It involved 17 males (56.7%) and 13 females (43.3%). Eight patients were phakic (26.7%), and 22 were pseudophakic (73.3%). The mean duration prior to silicone removal was 5.70 ± 2.17 months.

The preoperative BCVA mean was 1.00 ± 0.18 (LogMAR unit), and it exhibited a significant improvement postoperatively to 0.78 ± 0.15 (*p*-value < 0.001). In addition, postoperative IOP decreased significantly (from 15.33 ± 3.08 mmHg to 13.83 ± 2.52 mmHg*; p-*value < 0.001).

### Microperimetry data

The mean preoperative overall RS was 5.04 ± 2.06 dB, while the mean postoperative total RS was 6.38 ± 2.34 dB (*p*-value < 0.001). There was a postoperative significant elevation in the RS of each ring: inner (from 4.24 ± 2.45 to 5.89 ± 2.57 dB), middle (from 4.68 ± 2.06 to 6.23 ± 2.43 dB), and outer (from 5.13 ± 2.15 to 6.44 ± 2.34 dB) postoperatively (*p*-value < 0.001, < 0.001, and 0.002) consecutively.

### OCTA data

**Fig. 1:**
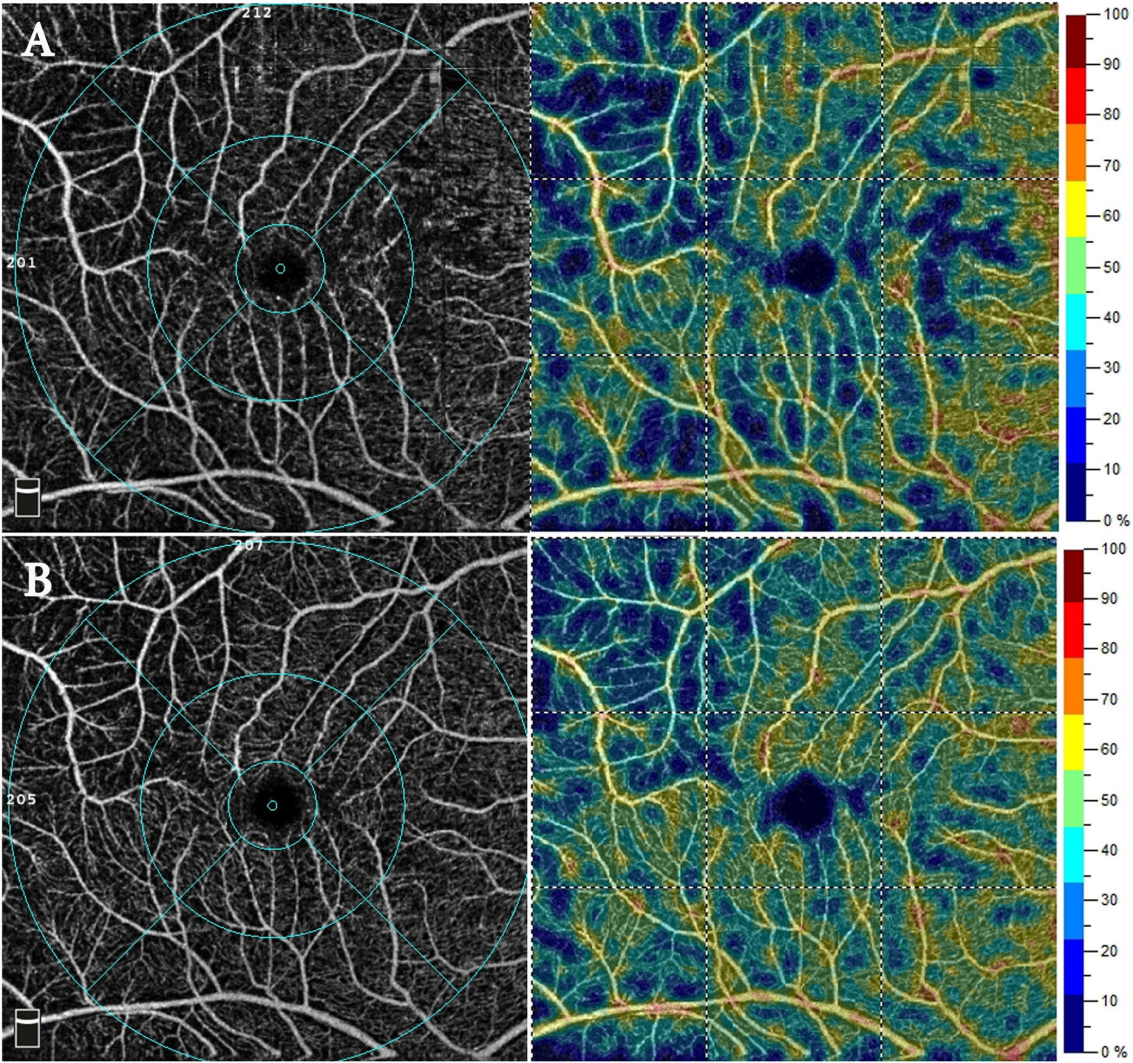
OCTA scans of the SCP from patient number 8. **A** Preoperative scans showing decreased vascular density with areas of capillary dropouts. **B** Postoperative scans showing well-organized capillary plexus density

**Fig. 2:**
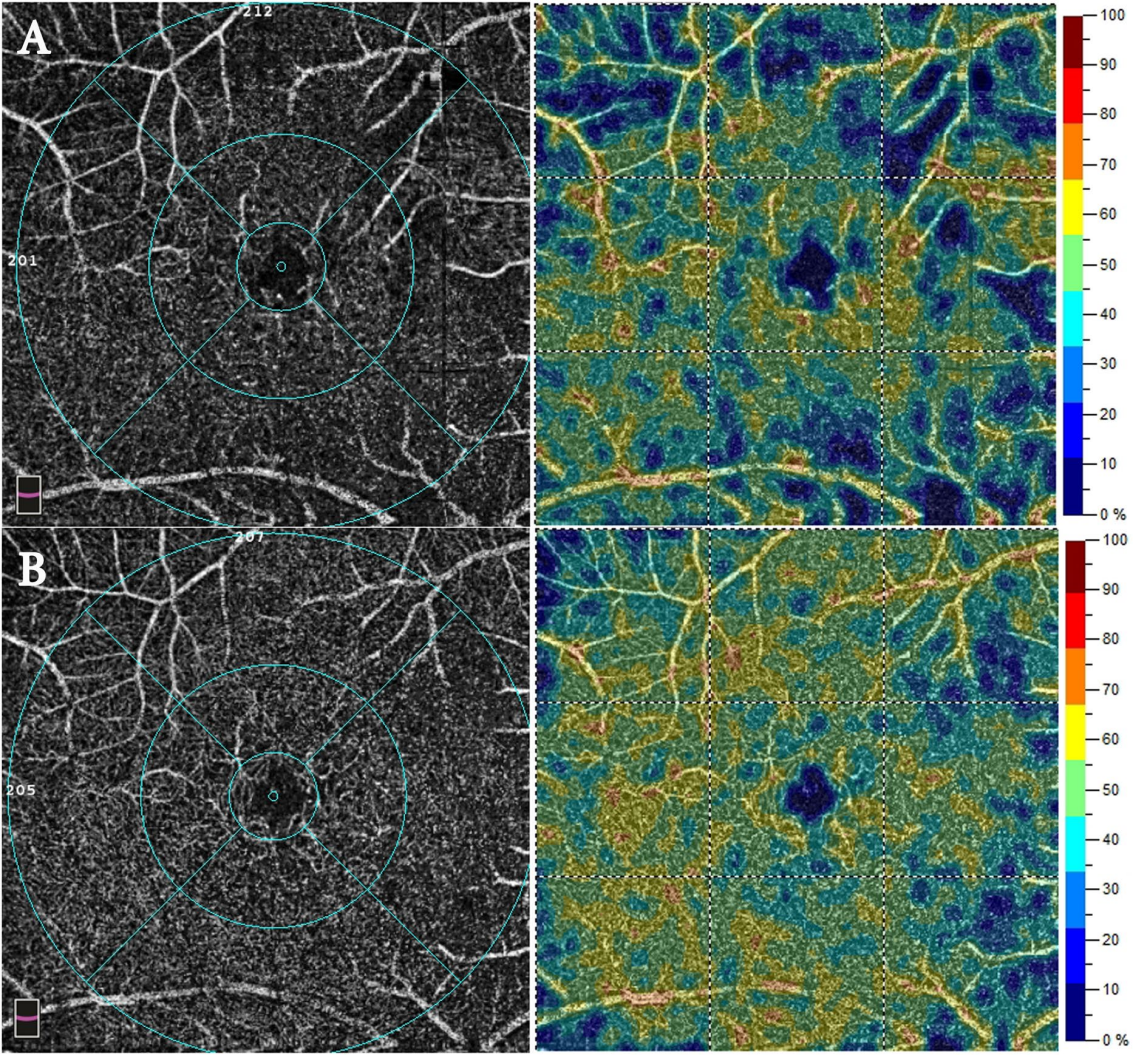
OCTA scans of the DCP from patient number 8. **A** Preoperative scans showing areas of capillary dropouts with capillary disorganization. **B** Postoperative scans showing improved capillary plexus density

Analysis of pre- and post-SO removal macular scans did not reveal any significant differences in the VD of the deep capillary plexus (DCP), choriocapillary plexus (CCP), and superficial capillary plexus (SCP). The mean size of the foveal avascular zone (FAZ) showed a non-significant decrease (*p*-value = 0.758). In contrast, the central foveal thickness (CFT) showed a significant postoperative increase with all values within the normal range of the age-matched group (*p*-value = 0.002), as shown in (Table 1 & Figs. 1, 2).

**Table 1:** Comparison between the postoperative and preoperative vascular density of the different macular layers by OCTA

	Preoperative	Postoperative	*p-*value
	Mean ± SD	Mean ± SD	
CFT (microns)	254.90 ± 29.63	265.50 ± 30.40	0.002*
FAZ (microns)	0.31 ± 0.47	0.28 ± 0.17	0.758
SCP foveal %	18.17 ± 5.60	18.63 ± 6.37	0.714
SCP parafoveal %			
Superior	38.39 ± 10.07	41.49 ± 8.57	0.180
Inferior	37.91 ± 8.72	40.00 ± 8.57	0.346
Nasal	36.08 ± 8.76	38.01 ± 7.41	0.354
Temporal	38.99 ± 7.35	36.63 ± 7.29	0.157
SCP perifoveal %			
Superior	40.42 ± 6.63	40.77 ± 8.01	0.823
Inferior	41.49 ± 5.64	41.09 ± 7.62	0.784
Nasal	41.72 ± 7.60	43.30 ± 8.22	0.318
Temporal	36.66 ± 5.49	36.98 ± 5.64	0.822
DCP foveal %	33.37 ± 8.36	37.03 ± 7.70	0.092
DCP parafoveal %			
Superior	44.48 ± 9.03	45.28 ± 7.66	0.652
Inferior	42.48 ± 8.58	43.01 ± 7.91	0.773
Nasal	44.11 ± 9.67	44.13 ± 8.58	0.995
Temporal	44.46 ± 8.54	43.70 ± 9.67	0.716
DCP perifoveal %			
Superior	39.53 ± 7.07	40.83 ± 7.04	0.490
Inferior	38.50 ± 7.52	39.80 ± 7.40	0.357
Nasal	38.59 ± 8.38	39.32 ± 7.15	0.697
Temporal	40.21 ± 7.20	42.05 ± 8.43	0.357
CCP %	2.04 ± 0.17	2.01 ± 0.17	0.163

*CFT* central foveal thickness, *SCP* superficial capillary plexus, *CCP* choriocapillary plexus, *SD* standard deviation

**Fig. 3:**
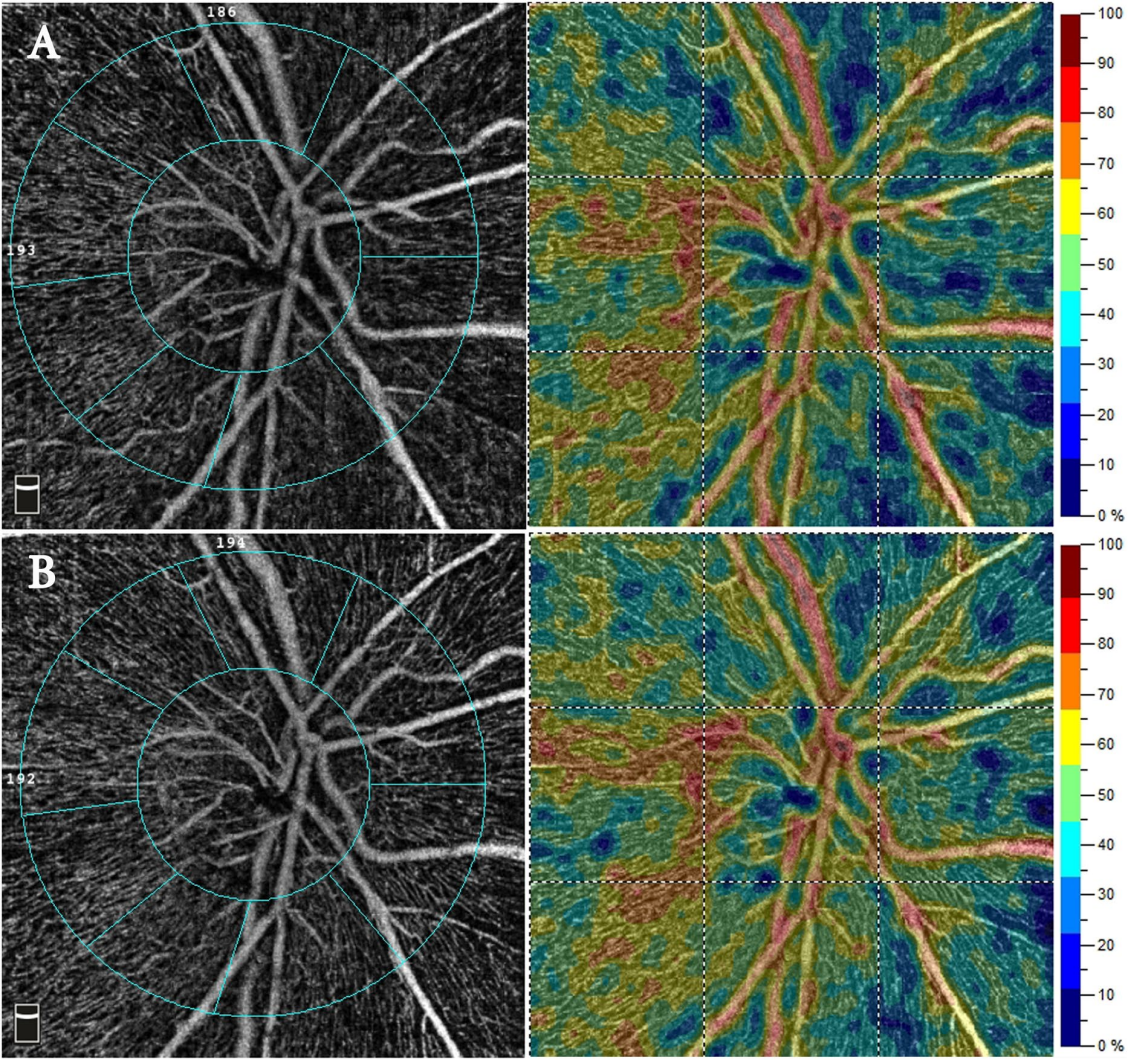
**A** Preoperative OCTA scans of the optic nerve VD. **B** Postoperative scans showing an increase in the VD in the capillary zones of the optic nerve from patient number 8

OCTA analysis of the optic nerve revealed a significant elevation in the postoperative VD of the whole image from 38.39 ± 5.53% to 41.31 ± 5.97% and peripapillary capillary plexus from 39.45 ± 7.33% to 43.04 ± 7.07*%* (*p-*value < 0.001 and 0.002, respectively)*,* as demonstrated in (Fig. 3).

#### Correlations between retinal sensitivity and vascular density

Using the Spearman correlation coefficient in the postoperative follow-up, there was a significant positive association between the total retinal sensitivity and CCP (*r* = 0.395, *p*-value = 0.031), as depicted in (Table 2).

**Table 2:** Correlation between percentage change of total retinal sensitivity with CFT, FAZ, SCP, DCP, CCP, and whole image optic disk

	Total retinal sensitivity percentage change	
	*R*	*p-*value
CFT	0.226	0.229
FAZ	− 0.027	0.888
SCP whole image	− 0.224	0.234
DCP whole image	0.069	0.716
CCP	− 0.057	0.764
Optic disk whole image	− 0.071	0.708

*SCP* superficial capillary plexus, *DCP* deep capillary plexus, *CFT* central foveal thickness, *CCP* choriocapillary plexus, *r* correlation coefficient

**Fig. 4:**
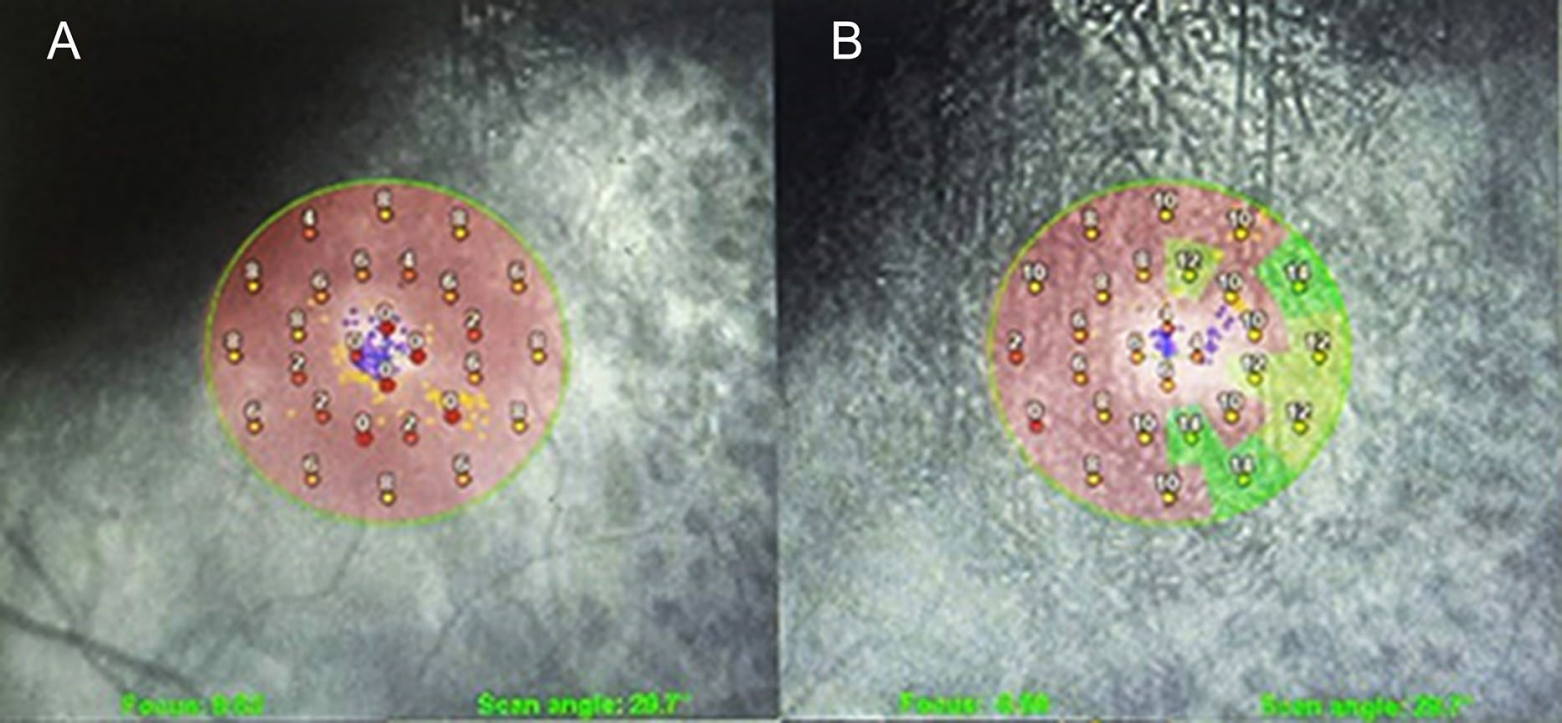
**A** Preoperative retinal sensitivity by microperimetry OPTOS polar 3–12° test of the left eye of patient number 11 with mean retinal sensitivity of 4.5 dB. **B** Postoperative retinal sensitivity of the same patient with a mean retinal sensitivity of 8.6 dB

When comparing the pre and postoperative BCVA with microperimetry (Fig. 4) and OCTA data of the macula and optic nerve, the only significant correlation observed was with the preoperative SCP foveal vascular density (*r* = 0.432, *p*-value = 0.017).

However, there were no statistically significant correlations between the duration of SO tamponade and the retinal sensitivity or the vascular density of OCTA macular layers and optic nerve head parameters.

The change in values between the preoperative and postoperative measurements was determined through a detailed examination of microperimetry and OCTA data. The total RS was 33.76 ± 50.68 dB, CFT change was 4.39 ± 7.25 microns, and FAZ was 102.93 ± 240.78 microns. The percentage change in the VD of SCP was 11.66 ± 15.23%, the DCP was 12.92 ± 20.33%, and the CCP was − 1.53 ± 6.65%. The VD of the whole image of the optic disk was 7.91 ± 9.42%.

Using Spearman correlation, there was a positive correlation between the percentage change in total RS with the percentage change in CFT and the DCP’s vascular density (*p* = 0.229 and *p* = 0.716, respectively), with no statistical significance. In contrast, there was a negative correlation between the total RS with FAZ, the VD of SCP, CCP, and the whole optic disk image (*p* = 0.888, 0.234, 0.764, and 0.708, respectively), with no statistical significance (Table 2).

## Discussion

This observational study utilized OCT, OCTA, and microperimetry to analyze the structural, vascular, and functional effects of silicone oil endotamponade on the optic nerve and macula. To our knowledge, this is the first study to determine the correlation between all these factors, attempting to elucidate the frequently unexpected visual outcomes following successful retinal detachment surgeries.

All our patients had macula-off rhegmatogenous retinal detachment, with successful reattachment following PPV with SO. Subsequently, there was a significant improvement in vision following SO removal.

In our pilot study conducted in 2018, we employed microperimetry to evaluate retinal sensitivity both before and after the removal of SO. We aimed to investigate the impact of SO on the retina. Interestingly, our findings indicated that irrespective of the duration of SO tamponade and the removal of SO led to a significant improvement in overall retinal sensitivity. Additionally, the sensitivity at the middle, inner, and outer rings of the retina also showed improvement [16]. In the current study, there was also a significant increase in the retinal sensitivity of each ring (middle, inner, and outer) postoperatively (*p*-value < 0.001, < 0.001, and 0.002 respectively).

In our study, while the FAZ area did not change significantly, the CFT showed a significant increase post-SO removal. This finding is consistent with previous studies, which demonstrated that the decrease in central macular thickness due to SO removal following surgical treatment of RRD is reversible, and CFT increases after SO removal. The decrease in thickness can be attributed to various mechanisms, including the toxic impact on ILM, the mechanical impact on the layers of the inner retina, or the alteration of the retinal environment due to the oil’s hydrophobicity [3, 17, 18].

Although our study failed to identify a correlation between the FAZ area in RRD and BCVA, Woo et al.’s study showed that both the superficial and deep FAZ areas in cases with macula-off RRD were negatively associated with BCVA during the first 2 months after RRD repair. In addition, the deep FAZ area was larger in macula-off RD eyes compared to macula-on RRD eyes. They hypothesized that the DCP might be more susceptible to hypoxia and that the expansion of the deep FAZ area might be a sign of macular ischemia that is severe. However, Sato et al. and Hong et al. found no association between postoperative BCVA at 1, 3, and 6 months following vitrectomy in macula-off RRD and the superficial or deep FAZ area [10, 19].

The aforementioned study focused exclusively on the changes in the FAZ area during the period after vitrectomy and did not investigate the period following silicone oil (SO) removal. It is noteworthy that the FAZ area in our study did not demonstrate significant changes pre- and post-SO removal and did not exhibit any correlation with the BCVA. We can infer that the SO tamponade was not responsible for the changes that may have been observed in these studies.

OCTA allows non-invasive imaging of the macular and optic nerve perfusion. Given the unexplained changes in visual acuity changes post-SO removal that did not correlate with the significant improvement in retinal sensitivity, our study aimed to utilize OCTA to identify any potential contributing vascular changes.

In our case series, we found no significant changes in the macular VD. However, we observed a significant increase in the postoperative vascular density of the whole image and peripapillary radial capillary plexus (*p*-value < 0.001 and 0.002), respectively, following SO removal.

Numerous studies have revealed that SO tamponade leads to both a reduction in outer retinal thickness and VD [20–22]. Nevertheless, few studies have evaluated the effect of SO removal on macular perfusion [23, 24]. In Prasuhn et al. [23], SO removal did not affect retinal perfusion values, with only a significant increase in the CCP postoperatively. Lee et al. reported no significant difference in VD following SO removal and 6 months following. Bayraktar et al. conducted an OCTA study to examine the superficial and DCP of the retina before and after SO removal. They found that the values of VD remained consistent throughout the study period [25].

With regard to the significant increase in the VD of the whole optic nerve image and in peripapillary radial plexus following SO removal, Wang et al. also detected an elevation in peripapillary capillary density following SO removal (most noted in the superior hemifield). They suggested that the use of SO tamponade could potentially impact peripapillary blood flow through capillary compression [26]. In our study, despite the strict exclusion of patients with increased IOP, a significant decrease in IOP following SO removal was still observed, which may have contributed to improved optic nerve perfusion. This finding aligns with the study conducted by Chen and colleagues, who concluded that peripapillary VD elevated following the decrease in IOP. However, they reported only mild changes in IOP, which did not influence macular microvascular parameters. It is worth noting that their study is different from ours as it was conducted on patients with ocular hypertension [27].

In our study, a substantial improvement in BCVA was observed following SO removal. However, no significant correlations were identified between BCVA and any of the microperimetry or OCTA data, except for VD of the fovea in SCP preoperatively. Despite a significant increase in retinal sensitivity, no significant correlations were found between preoperative, postoperative, or percentage change in retinal sensitivity and macular vascular density.

In order to eliminate confounding factors, none of our patients had associated cataract extraction during the SO removal. Moreover, any patient who developed high IOP with optic neuropathy was excluded.

The improved retinal sensitivity on microperimetry in our pilot research, as well as in another investigation comparing SO with gas tamponade in cases of retinal detachment, did not show a significant correlation with BCVA, which also exhibited a notable improvement. The potential impact of SO tamponade on ocular functioning, particularly in resolving spatial patterns for sensitivity and acuity in microperimetry, was utilized to elucidate this finding [16, 18].

Other studies also correlated macular VD in eyes undergoing PPV with SO tamponade and BCVA and did not report significant correlations. [22, 28, 29]. Therefore, we can conclude that changes in visual acuity following anatomically successful PPV are not likely attributed to a potential deleterious effect of SO tamponade. However, attributing the improvement in BCVA and retinal sensitivity solely to the SO removal is not completely valid, as it may be one of several factors, such as the natural healing process following anatomical reattachment of the retina, that contributed to this finding.

Wang et al. [11] and Hong et al. [19] also could not find significant correlations with the SCP or DCP VD. Nevertheless, they found a significant correlation with CCP VD, suggesting that the restoration of the choriocapillaris and deep retinal plexus is substantially associated with functional consequences. It is important to note that, unlike our study, these studies only evaluated patients during SO tamponade and did not follow-up with patients.

The duration of SO tamponade did not demonstrate any significant correlation with retinal sensitivity or vascular density of OCTA macular layers and optic nerve head parameters, which is consistent with findings from previous studies [23, 30, 31]. Lee et al. [24] found a significant association between the duration of SO tamponade and DCP VD. They hypothesized that the detrimental effects of SO on retinal tissues caused a drop in macular VDs at a particular time point following surgery [32].

In our study, the duration of silicone oil (SO) tamponade varied from 3 to 11 months, with a mean of 6 months, and yet, no correlation was found with best-corrected visual acuity (BCVA), allowing us to dismiss the potentially harmful effect related to this factor.

Furthermore, the total retinal sensitivity was positively and significantly linked with CCP (*r* = 0.395, *p*-value = 0.031). The choroid is considered the principal source of oxygen to the retina, and thus, altering its circulation can affect both the RPE and outer retina, leading to a disturbance in visual function [33]. According to Prasuhn et al., this substructure should not be disregarded when examining morphological changes [23].

The reason behind the increase in retinal sensitivity upon removal of the silicone oil remains unclear. Adverse effects such as cataracts, silicone emulsification, and secondary glaucoma can worsen after the removal of SO. These effects might significantly influence vision and retinal sensitivity. Nevertheless, we categorically excluded these individuals from our investigation.

## Conclusion

To our knowledge, this is the only study evaluating the functional, structural, and vascular effect of SO tamponade using OCTA and microperimetry. The main aim was to elucidate the factors contributing to changes in visual acuity following successful RRD surgery. However, this study has some limitations. Our postoperative analysis was conducted only 1 month following SO removal, and a long-term comparison at 6 and 12 months might have provided more conclusive results. Another limitation was the absence of the fellow eye as a control group. Even though we did not find a significant correlation with the duration of the endotamponade, unifying or comparing similar durations of tamponade may add more definitive conclusions on the potential adverse effects of SO. While the benefits of using SO have long been established, the mechanism by which it affects vision and whether it is attributed to changes in optic or macular perfusion is yet to be defined.

## Data Availability

All the data used and/or analyzed during the current study are available and can be presented by the corresponding author upon a reasonable request.
